# Pacific Coast Association of Examiners

**Published:** 1895-04

**Authors:** 


THE Pacific Coast Association of Examiners was organized at at Portland, Oregon, January 30, 1895. Dr. F. M. Bell presided, and Dr. H. W. Coe, of Portland, acted as Secretary. Sixty-six physicians became members, representing Oregon, Washington, Montana, Idaho, British Columbia, California and Utah. Dr. Richmond Kelley, of Portland, Ore., was elected President, and the following were elected Vice-Presidents for their respective States: Dr. L. M. Sims, of Kalama, Washington; Dr. W. J. McGuigan, Van Couver, British Celumbia; Dr. F. D. Bullard, Los Angeles, California; Dr. C. K. Cole, of Helena, Montana, Dr. C. L. Sweet, Boise, Idaho; Dr. S. C. Baldwin, Salt Lake City, Utah; Dr. J. D. Fenton, Portland, Oregon. Dr. H. McCoe, of Portland, was elected Secretary. The annual dues were fixed at $2. The *Medical Examiner*, of New York, was made the official organ. The question of ''careless examinations'' was discussed, and, upon motion of Dr. Amos, of Portland, a provision was placed in the by-laws, calling upon the executive committee to summarily dismiss any member, who, upon sufficient evidence, should appear to have acted either in a careless manner, or in collusion with the agent, to the loss of the company, and to the detriment of more rigid examiners. Dr. H. W. Coe read a paper on Over-weight, which is published in the February number of the *Medical Examiner*.

Why can we not have an Association of Life Insurance Medical Examiners iu Texas? The JOURNAL suggests that one be formed at the Dallas meeting of the Texas State Medical Association this month.

